# The Interaction of Facial Expression and Donor-Recipient Eye Contact in Donation Intentions: Based on the Intensity of Emotion

**DOI:** 10.3389/fpsyg.2021.661851

**Published:** 2021-08-17

**Authors:** Zelin Tong, Mengjie Yi, Wenting Feng, Yinghao Yu, Diyi Liu, Jixuan Zhang

**Affiliations:** ^1^Management School, Hainan University, Haikou, China; ^2^School of Business Administration, Zhongnan University of Economics and Law, Wuhan, China; ^3^HNU-ASU Joint International Tourism College, Hainan University, Haikou, China

**Keywords:** happy facial expression, sad facial expression, eye contact, emotional intensity, donation intention

## Abstract

Both happy and sad facial expressions of recipients are frequently used in charity advertisements. However, the relative effectiveness of these two types of facial expressions has been found paradoxical in the past. In this study, we examine when happy facial expression can more effectively increase donation intentions of consumers and when vice versa. Specially, we propose that eye contact between a donor and a potential recipient may moderate the relative effectiveness of happy and sad facial expressions, and further explain the interaction effect from the perspective of emotional intensity. Results from two experiments suggest that, when donor-recipient eye contact is present, consumers tend to have stronger emotional intensity, and, in turn, show higher donation intentions when the recipient is with a happy rather than sad facial expression. In contrast, when the eye contact is absent, consumers may show stronger emotional intensity and donation intentions toward the charity advertisement with a recipient showing sad rather than happy expression.

## Introduction

Donations from individuals were estimated at 286.65 billion dollars in 2018, outpacing the donation from foundations and corporations and currently growing at a higher rate than the other sources of donation (Hodson, 2018). In order to encourage individuals to make donations, charity organizations often use fundraising advertisements, showing facial expressions of potential recipients (Cao and Jia, 2017). Prior research (Ekman et al., 1972; Keltner et al., 2003; Small and Verrochi, 2009) examined the relative effectiveness of different types of facial expressions, mainly focusing on the influence of happy and sad expressions. Some studies (Coke et al., 1978; Bagozzi and Moore, 1994; Zemack-Rugar and Klucarova-Travani, 2018) suggest that happy rather than sad facial expression can more effectively encourage consumers to make donations, considering that happy facial expression may provide donors with a desirable prospect of their donations, such as the prospect that the recipients will be able to get out of their current dilemma. In the meantime, some other research (Small and Verrochi, 2009; Genevsky and Knutson, 2015) provides contradictory findings, indicating that sad facial expression is more likely to trigger sympathy of donors, and, hence, sad rather than happy facial expression can be more effective in increasing willingness of consumers to donate.

A question arises as of when showing happy facial expression is more effective than sad expression, and when vice versa. To explain the mechanism underlying these contradictory findings, some research (Obermiller, 1995; Faseur and Geuens, 2010; Cao and Jia, 2017) has investigated potential moderators of the ambivalent relative effectiveness of happy and sad facial expressions. Cao and Jia (2017) have examined the moderating roles of the characteristics of donors, such as involvement with charities. Specifically, less involved donors are more likely to be motivated by sad (vs. happy) facial expression, since sad facial expression conveys a stronger message that the recipients are in need (Obermiller, 1995; Faseur and Geuens, 2010). Some studies (Yan et al., 2010; Sharma and Morwitz, 2016; Reed and DeScioli, 2017) also examined the interaction effect of facial expression and charity advertisement messages, such as the regulatory focus of the advertising message (Zemack-Rugar and Klucarova-Travani, 2018). Findings from these studies (Yan et al., 2010; Reed and DeScioli, 2017) showed that when a charity advertisement shows happy facial expression of a recipient, consumers tend to have higher donation intentions when the advertising message is promotion focused rather than prevention focused; whereas when the recipient shows sad facial expression, regulatory focus on the advertising message has no significant effect (Sharma and Morwitz, 2016).

However, the existing studies (Ravaja et al., 2004; Mas and Moretti, 2009; Liang et al., 2016) on charity advertisement have not fully considered the impacts of the characteristics of the social interaction between donors and potential recipients, which may help increase the persuasiveness of a charity request (Ravaja et al., 2004), concern of self-reputation of donors (Mas and Moretti, 2009), the trigger for strong emotions (Liang et al., 2016), and, in turn, enhancement of their donation intentions. Considering that eye contact is one of the most important avenues of social interactions between a donor and a recipient, we argue that the presence of donor-recipient eye contact may influence donation intentions of consumers. In practice, charity organizations also have taken different approaches; while some charity organizations, such as Waterdrop Fundraising, may choose to show the eyes of recipients in their campaigns, some other organizations (e.g., Tencent Welfare and Alipay’s Donation platforms) may decide to intentionally cover the eyes of potential recipients for their privacy. Some research (Ernest-Jones et al., 2011; Oda et al., 2011; Ekström, 2012) has investigated the effects of eye contact with eye images on consumer behaviors. For example, Oda et al. (2011) found that even an eye-like painting could also enhance expectation of consumers of a good reputation. And displaying eye images could cause people to engage in cooperative behavior (Ernest-Jones et al., 2011). Furthermore, Ekström (2012) suggested that eye contact can effectively enhance donation intentions. Specifically, he found that when viewing a drawing of a pair of eyes, consumers tend to show higher donation intentions and altruistic behaviors. Fathi et al. (2014) also found that eye contact with eye images could significantly increase average donations. Nevertheless, there is still a lack of in-depth insight into the potential interaction effect between the presence of eye contact and facial expressions.

In this study, we aim to investigate how two different facial expressions (i.e., happy and sad expressions) influence donation intentions of consumers and the moderation effect of the presence or absence of eye contact and the mediation effect of emotion intensity. First, we investigated the effect of two different facial expressions on donation intentions of consumers and the moderation effect of the presence or absence of eye contact. Specifically, we propose that, given a charity advertisement, happy rather than sad facial expression of a recipient can increase donation intentions of consumers more effectively when the donor–recipient eye contact is present; whereas sad facial expression may be more effective in enhancing donation intentions when the eye contact is absent. Second, we illustrate the mechanism underlying the moderation effect of donor–recipient eye contact from the perspective of emotion intensity of consumers aroused by a charity advertisement. We propose that the interaction effect of happy facial expressions and donor–recipient eye contact on donation intention is mediated by the intensity of the positive emotions of the donor, while the negative emotions of the donor mediate the interaction effect of sad facial expressions and donor–recipient eye contact on donation intention. In this study, we used two between-subjects designs to testify our hypotheses.

The following sections are organized as follows: firstly, we provided a review of the existing literature on the impacts of the facial expression of recipients, donor-recipients eye contact, and emotion intensity on donation behaviors. We then tested our hypotheses with two experiments. Finally, we discuss the theoretical contributions, managerial implications, and directions for future research.

## Literature Review

### The Impacts of Facial Expressions on Donation Intentions

Both happy and sad facial expressions are frequently used in charity advertisements, largely because facial expressions can function as an effective tool in enhancing the persuasiveness of the charity advertisement, leading to favorable responses, such as increased donations (Tidd and Lockard, 1978; Solomon et al., 1981; Cao and Jia, 2017). Drawing inferences from the theory of emotional contagion, facial expression may elicit vicarious emotion of observers (Hatfield et al., 1993) and, hence, have significant influence on donation intentions of consumers (Hackenbracht and Tamir, 2010).

Prior studies (e.g., Forest et al., 1979; Dyck and Coldevin, 1992; Zemack-Rugar and Klucarova-Travani, 2018; Goenka and Van Osselaer, 2019) provided ambivalent findings on the relative effectiveness of happy and sad facial expressions. On the one hand, some (Forest et al., 1979) suggest that happy facial expression can be more effective in motivating consumers to make donations, showing that happy facial expression can motivate individuals to help others since altruistic behavior is inherently pleasant and enables the duration of the positive mood. In a charity advertisement, happy facial expression of a recipient may induce positive emotions of donors, triggering their positive emotional experience (Dyck and Coldevin, 1992) and rendering higher donation intentions for the purpose of maintaining the positive emotion (Faseur and Geuens, 2010; Zemack-Rugar and Klucarova-Travani, 2018; Goenka and Van Osselaer, 2019). On the other hand, some studies (e.g., Van Doorn et al., 2015; Cao and Jia, 2017) indicate that sad facial expression can be more effective than happy expression. Prior research (Baumann et al., 1981; Cialdini et al., 1982) showed that a negative mood motivates people to alleviate the unpleasant feeling, driving individuals to engage in behaviors to alleviate the unpleasant feelings (Van Doorn et al., 2015; Cao and Jia, 2017). Findings from other studies (Burt and Strongman, 2005; Small and Verrochi, 2009; Baberini et al., 2015) also suggest that making donations can be an effective approach to help consumers cope with negative emotions. Given the above ambivalent findings on the effectiveness of happy and sad facial expressions, we further examined when happy facial expression can better increase donation intentions of consumers and when vice versa.

### Moderating the Role of Eye Contact

Eye contact is an effective cue that can facilitate pro-social behaviors (Vaish et al., 2017; Kelsey et al., 2018; Cañigueral and Hamilton, 2019). Individuals are more likely to take observers into account and commit to pro-social behaviors when they perceive themselves being observed (Soetevent, 2005; Alpizar et al., 2008). Ekström (2012) found that even subtle cues of being observed also affect pro-social behaviors, such as eye contact. Findings from prior research (Bateson et al., 2006; Francey and Bergmüller, 2012; Powell et al., 2012) indicate that the presence of eye contact can increase cooperative behavior (Bateson et al., 2006), refrain from littering (Francey and Bergmüller, 2012), and improve donation intentions (Powell et al., 2012). The presence of eye contact brings forth the feeling of being observed and, hence, enhance concern of donors of their self-reputation (Haley and Fessler, 2005). Second, eye contact can also affect how individuals process the information of facial expression of others (Adams and Kleck, 2005). Existing research (Argyle and Cook, 1976; Harmon-Jones and Segilman, 2001) has investigated how the presence of eye contact influences the efficiency of recognition of individuals of emotion information *via* facial expression. Specifically, the presence/absence of eye contact may trigger different types of motivation; while the presence of eye contact is likely to be associated with approach motivation, the absence of eye contact often relates to avoidance motivation (Argyle and Cook, 1976; Harmon-Jones and Segilman, 2001). Since positive (vs. negative) emotions are generally affiliated with approach (avoidance) motivation, the presence of eye contact may trigger stronger emotional responses when it is shown with matched facial expressions (Adams and Kleck, 2003, 2005). Hence, the presence of eye contact is likely to facilitate the processing of happy facial expression, whereas the absence of eye contact may enhance the processing of sad facial expression (Adams and Kleck, 2003). Third, the presence of eye contact can also influence emotional perception of consumers. As for emotional perception, Messinger et al. (2012) found that eye constriction intensity is positively associated with both smile intensity and cry intensity. Furthermore, prior research (Willis et al., 2011; Sutherland et al., 2017) revealed that the presence of eye contact also has an impact on social judgment of individuals, such as trustworthiness judgments. For example, Willis et al. (2011) found that eye contact influences trustworthiness judgments of individuals. When there is presence of eye contact instead of absence of eye contact, happy faces are perceived more trustworthy. The results are also revalidated by Sutherland et al. (2017). They found that happy expressions were considered notably trustworthy with facing directly.

Considering the potential impacts of eye contact on processing of individuals of facial expressions, we argue that the relative effectiveness of happy and sad expressions may be moderated by the presence of eye contact in a charity advertisement. When there is eye contact between a donor and a potential recipient in the charity advertisement, the donor may facilitate the processing of happy (vs. sad) facial expression, experience stronger positive emotion (Adams and Kleck, 2003), and consider that the recipient more likely to overcome obstacles (Kemp et al., 2013). Subsequently, potential donors may be inclined to make a donation to maintain these positive emotional experiences (Manucia et al., 1984). Hence, a recipient with a happy facial expression (versus a sad facial expression) increases donation intentions when the donor-recipient eye contact is present. In contrast, when the eye contact between a donor and a recipient is absent, the donor may facilitate the processing of a sad (vs. happy) facial expression and experience stronger negative emotions (Adams and Kleck, 2003). To alleviate the negative feelings, donors may tend to conduct prosocial behaviors, such as making donations (Cialdini and Kenrick, 1976; Kenrick et al., 1979). Hence, consumers may have higher donation intentions when viewing a recipient with a sad (vs. happy) facial expression. Thus, we propose the following hypotheses.

H1a: In the presence of donor–recipient eye contact in a charity advertisement, showing *happy* rather than sad facial expression of the recipient may more effectively increase donation intentions of consumers.

H1b: In the absence of donor–recipient eye contact in a charity advertisement, showing *sad* rather than happy facial expression of the recipient may more effectively increase donation intentions of consumers.

### The Mediating Role of Emotional Intensity

We explain the interaction effect between the presence of eye contact and two types of facial expressions and propose that emotional intensity of consumers aroused by a charity advertisement may serve as the underlying mechanism. Prior research (N’Diaye et al., 2009) suggested that when eye contact matches the underlying behavioral intention communicated by a facial emotional expression, emotion intensity can be enhanced. Furthermore, researchers reveal that eye contact moderates the judgment of emotion intensity in facial expression (Sander et al., 2007).

A number of studies (Batson et al., 1979; Adams and Kleck, 2003) demonstrate that, for both positive and negative emotions, emotional intensity can result in increased donation intentions, since stronger emotional intensity may lead to higher responsiveness to needs of others. For example, Batson et al. (1979) found that a higher intensity of positive emotions (e.g., happiness) increased responsiveness of individuals to needs of others, because the act of helping others is perceived to be happy and promoted the willingness to donate. Similarly, Adams and Kleck (2003) found that a higher intensity of negative emotions (e.g., sadness) also promoted responses to needs of others, because a higher intensity of sadness can trigger empathetic behavior of consumers toward the donation target, and, in turn, increase donation intention. Thus, we propose:

H2a: The interaction effect of happy facial expressions and donor-recipient eye contact on donation intention is mediated by the intensity of *positive* emotions of the donor.

H2b: The interaction effect of sad facial expressions and donor-recipient eye contact on donation intention is mediated by the intensity of the *negative* emotions of the donor.

## Methodology

### Pilot

Before Study 1, we did a pilot study to select the photo of the recipient, in which 52 participants were instructed to view two pairs of photos and evaluate the extent to which each photo conveyed happy and sad emotions. We took pictures of each recipient (i.e., a boy and a girl), showing a happy or sad expression. The results of the pretest show that both photos with happy expression are considered happier (*M*boy = 6.00, *SD*boy = 1.05; *M*girl = 5.7, *SD*girl = 1.02) than the pictures with sad expression (*M*boy = 2.06, *SD*boy = 0.87; *M*girl = 2.40, *SD*girl = 0.96, and *p* < 0.001). The photos with the highest evaluations on happiness and sadness were chosen as stimuli. According to the research of Small and Verrochi (2009), the gender of child does not influence donation intentions; thus, we only consider one condition in the following research.

### Study 1

The aim of Study 1 is to examine the interaction effect of facial expression and the presence of eye contact on donation intentions of consumers. Study 1 is a 2 (happy vs. sad facial expression) × 2 (eye contact present vs. absent) between-subject design.

#### Procedure

The procedure of the experiment is as follows. First, we presented the participants a photo of a boy in need of help (i.e., a recipient) and asked them to identify the facial expression of the recipient. Second, they were instructed to read a donation campaign (as shown in Appendix), which includes a brief description of the recipient and the photo presented previously. After reading the description, the participants were asked to finish a questionnaire, which measured their donation intention, involvement with the campaign, and perceived credibility of the donation campaign.

#### Participants

We recruited 201 participants (113 females) to participate in the experiment in exchange for 1 dollar each. Of the participants, 48.3% were between the age of 19 and 39, and 39.3% were between 30 and 39 years old. As for monthly income, 80.1% of the participants earned less than US$1,540, and 4.5% of them earned more than US$3,080 per month.

#### Stimuli and Instruments

The stimuli were chosen from a pilot, which is a boy with happy or sad facial expression. Facial expression is manipulated as follows: for the participants in the happy facial expression group, the recipient in the photo presented is with a smiling face; whereas, for those in the sad facial expression group, the recipient in the photo is with a crying face. Moreover, the presence of eye contact is manipulated with the following procedure: in the condition in which eye contact is absent (vs. present), we covered the eyes of the recipient in the photo.

As for measurement, we measured the involvement of the participants with the task and perceived credibility of the donation campaign, as well as their donation intentions. Involvement with the donation campaign was measured with items adapted from Gurhan-Canli and Batra (2004), including “*I strongly believe in the description of the recipient in the campaign*,” “*I fully accept the description of the victim in the above materials*,” and “*I believe that the description of the victim in the above materials is reliable*” (*Cronbach’s*α = 0.819). Perceived credibility was assessed with the measurements adapted from Gurhan-Canli and Batra (2004), including “*after reading the above materials, I feel that the description of the victim is very realistic*” and “*I have read all the above materials very carefully*” (*Cronbach’s*α = 0.783). Involvement with the donation campaign and perceived credibility are measured on a seven-point Likert-like scale, ranging from 1, “strongly disagree,” to 7, “strongly agree.” To measure donation intentions, we asked the participants to elicit how much they were willing to donate, with the donation amount ranging from US$0 to US$9. We also asked the participants about whether the mosaic had a negative influence on the judgment of expression.

#### Statistical Analysis

In this section, we used IBM SPSS Statistics 24 and JASP to do all the statistical data analysis.

*Manipulation check.* On the manipulation check, compared with the participants in the sad facial condition, those in the happy facial expression condition have significant higher evaluations on the perceived happiness of the recipient [*M*happy = 6.06, *M*sad = 1.78, *t*(103) = 17.08, and *p* < 0.001] and lower evaluations on the perceived sadness [*M*happy = 5.94, *M*sad = 2.02, *t*(94) = 15.00, and *p* < 0.001]. Furthermore, the presence of eye contact has no significant influence on the perception of the participants of the facial expression of the recipient. Specifically, compared with those in the eye contact presence condition, the participants in the eye contact absence condition have no significant difference on their evaluations on the perceived happiness [*M*presence = 6.05, *M*absence = 5.94, *t*(99) = 0.46, and *p* = 0.65] or the perceived sadness [*M*presence = 1.77, *M*absence = 2.02, *t*(98) = −0.97, and *p* = 0.33] of the recipient. Also, the participants perceived the mosaic had no influence on expression recognition between the presence of eye contact when there was happy expression [*M*presence = 5.62, *M*absent = 5.14, *t*(99) = 1.62, and *p* = 0.11] and when there was sad expression [*M*presence = 5.54, *M*absent = 5.63, *t*(98) = −0.33, and *p* = 0.75].

**FIGURE 1:**
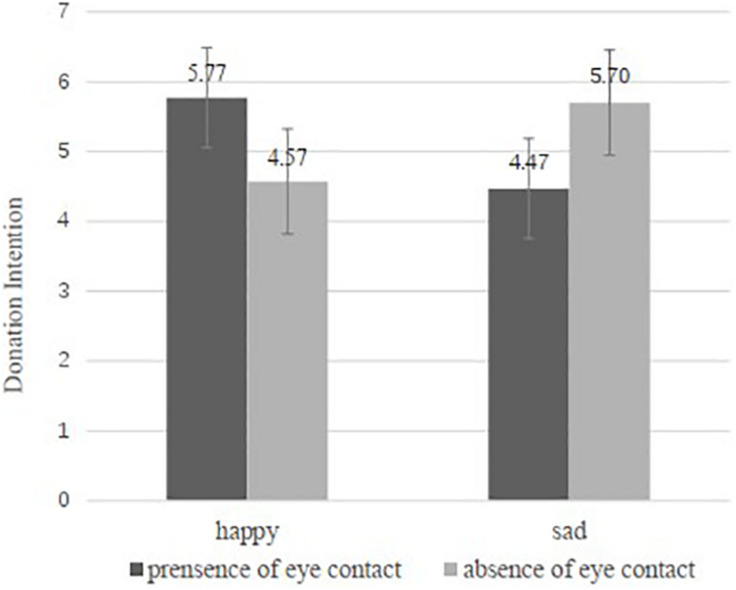
Effect of facial expression and eye contact on donation amount in Study 1.

*Donation intention*. We then conducted a two-way ANOVA of facial expression and eye contacts on the donation intentions of the participants. The results indicate that the main effects of facial expression [*F*(1,197) = 0.049, *p* = 0.825, and partial η^2^ = 0.000] and eye contact [*F*(1,197) = 0.002, *p* = 0.968, and partial η^2^ = 0.000] are not significant. However, there is significant interaction effect of the facial expression and the presence of eye contact [*F*(1,197) = 10.851, *p* = 0.001, and partial η^2^ = 0.052]. Specifically, as shown in Figure 1, in the presence of eye contact condition, the participants donated significantly higher amounts when the recipient in the photo was with happy facial expression rather than sad one (*M*happy = 5.77, *M*sad = 4.47, and *p* = 0.013). In contrast, in the absence of eye contact condition, the participants made fewer donations when the recipient in the photo was happy rather than sad (*M*happy = 4.57, *M*sad = 5.70, and *p* = 0.032). Furthermore, the results of Bayes factor analysis also provided strong evidence for our hypotheses (BF_10_ = 27.570). H1a and H1b are supported. In addition, we also examined the potential confounding effects of involvement and credibility. The results of two-way ANOVA indicate that the participants in the four conditions show no significant difference on involvement with the donation campaign among the four groups [*F*(3,197) = 1.74, *p* = 0.159, and partial η^2^ = 0.026], but there is significant difference on perceived credibility of the campaign among the four groups [*F*(3,197) = 2.68, *p* = 0.048, and partial η^2^ = 0.039].

### Study 2: Mediation Effect of Emotional Intensity

In Study 2, we aim to investigate the psychological mechanism underlying the interaction effect of facial expression and the presence of eye contact with 2 (happy vs. sad facial expression) × 2 (eye contact presence vs. absence) between-subject design. Specifically, we examine the mediating role of emotional intensity of the participants, including both positive emotional intensity and negative emotional intensity.

#### Procedure

The procedure of Study 2 is the same in Study 1. First, the participants were showed a photo of a boy (i.e., a recipient) with happy or sad facial expression. Then, the participants were told to read a donation campaign. After reading the donation campaign, we measured the emotional intensity, donation intentions, involvement with the campaign, and perceived credibility of the donation campaign of the participants.

#### Participants

A total of 213 participants (115 females) were recruited to participate in the experiment in exchange for 1 dollar each. Among the participants, 46.5% were with the age between 19 and 29, 40.8% were between 30 and 39 years old. As for monthly income, 23.% earned between US$308 and US$770, 62.9% earned between US$771 and US$1,540, and 10.3% earned more than US$1,540.

#### Stimuli and Instruments

The stimuli of Study 2 are the same with the stimuli in Study 1. The measurements of donation intentions, involvement with the campaign (*Cronbach’s*α = 0.826), and perceived credibility (*Cronbach’s*α = 0.750) are the same as in Study 1. Furthermore, we also measured the emotional intensity of the participants. The measurement of emotional intensity was adapted from Bachorowski and Braaten (1994), including both positive and negative emotional intensities, and each was measured with three items: positive emotional intensities include very happy, ecstatic, and enthusiastic (*Cronbach’s*α = 0.786), and negative emotional intensities contain very sad, finding it difficult to breathe, and depressed (*Cronbach’s*α = 0.849).

#### Analysis

IBM SPSS Statistics 24 and JASP were used to do all the statistical data analysis.

##### Manipulation check

The results of the manipulation check shows that the participants in the happy (vs. sad) condition are more likely to perceive the recipient happy rather than sad [*M*happy = 5.25, *M*sad = 1.64, *F*(1,211) = 282.191, *p* < 0.001, and η^2^ = 0.572]. Specifically, in the presence of eye contact condition, the participants in the happy facial expression group perceived the facial expression happier than those in the sad group (*M*happy = 5.53, *M*sad = 1.87, and *p* < 0.001), which is consistent in the absence of eye contact condition (*M*happy = 4.96, *M*sad = 1.42, and *p* < 0.001). In addition, the presence or absence of eye contact has no significant influence on facial expression recognition (*M*presence + happy = 5.53, *M*absence + happy = 4.96, and *p* = 0.062; *M*presence + sad = 1.87, *M*absence + sad = 1.42, and *p* = 0.130).

##### Donation intentions

**FIGURE 2:**
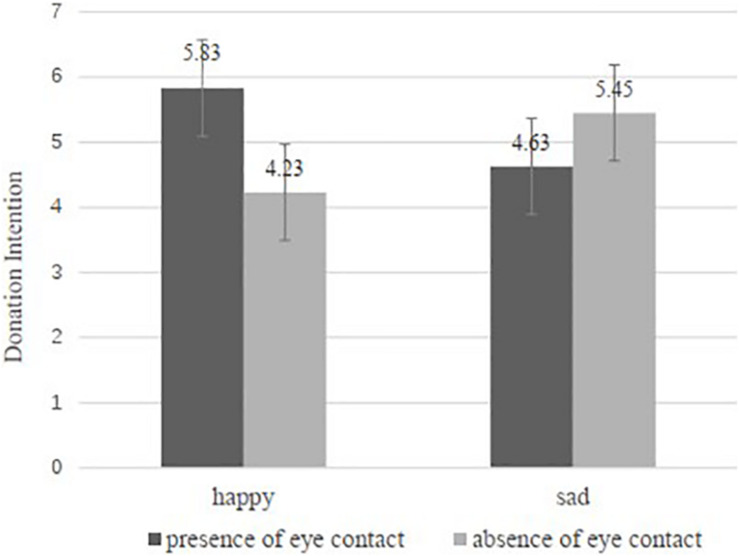
Effect of facial expression and eye contact on donation amount in Study 2.

Then we conducted a 2 (facial expression: happy vs. sad) × 2 (eye contacts: with eye contacts vs. without eye contacts) between-subjects ANOVA on donation intentions. The results of the two-way ANOVA indicate significant interaction effect of facial expression and eye contact [*F*(1,209) = 10.556, *p* = 0.001, and partial η^2^ = 0.048], yet no significant main effects of facial expression [*F*(1,209) = 0.001, *p* = 0.972, and partial η^2^ = 0.000] or the presence or absence of eye contact [*F*(1,209) = 1.092, *p* = 0.297, and partial η^2^ = 0.005]. Furthermore, the results of Bayes factor analysis also provided strong evidence for our hypotheses (BF_10_ = 21.932). As shown in Figure 2, in condition where eye contact was present, the participants donated more when the recipient was displayed with happy instead of sad facial expression (*M*happy = 5.83, *M*sad = 4.63, and *p* = 0.024); whereas, in condition where eye contact was absent, the participants donated less when the recipient was happy rather than sad (*M*happy = 4.23, *M*sad = 5.45, and *p* = 0.022). The results revalidate H1a and H1b.

##### Mediation analysis

Moreover, we tested the mediation effect of emotional intensity with mediated moderation analysis, using the bootstrapping procedure suggested by Hayes (2013). We conducted mediation analysis with Model 7 and a bootstrap sample of 5,000. The results show that, while the direct effect of facial expression is not significant [β = −0.731, *SE* = 0.483, 95%, and CI = (−1.684, 0.221)], emotional intensity has significant moderated mediation effect [β = 0.267, *SE* = 0.190, 95%, and CI = (0.018, 0.810)]. The mediation effect of emotional intensity is both significant when eye contact is present [β = 0.607, *SE* = 0.261, 95%, and CI = (0.147, 1.195)] and when eye contact is absent [β = 0.874, *SE* = 0.383, 95%, and CI = (0.181, 1.695)]. The results of Study 2 suggest that emotional intensity mediates on the interaction effect of facial expression and eye contact on the donation amount. H2a and H2b are supported.

## Discussion

Two experiments above supported our four hypotheses. First, the results of Study 1 revealed a significant interaction effect of facial expression and eye contact on the willingness to donate, supporting H1a and H1b. Specifically, in the presence of eye contact, the participants have higher willingness to donate when the recipient presented in the donation campaign is happy (vs. sad); however, in the absence of eye contact condition, the participants show higher disinclination to donate when the recipient is with sad (vs. happy) facial expression. Second, the results of Study 2 suggest that emotional intensity mediates on the interaction effect of facial expression and eye contact on the donation amount. Both H2a and H2b are supported. To be specific, happy expressions resulted in more intense positive emotions than sad expressions with the presence of eye contact between the donor and the donee, which was associated with a higher donation amount. In the absence of eye contact condition, sad expressions resulted in more intense negative emotions than happy expressions, which was associated with a higher donation amount.

## Conclusion

This study examines the moderation effect of presence of eye contact on the relative effectiveness of happy and sad facial expressions of a recipient, as well as the mechanism underlying this effect. The results from the two experiments suggest significant interaction effect between facial emotion expression and eye contact of donor-recipient on donation intentions of consumers; when eye contact between a donor and a recipient is present, consumers tend to have higher donation intentions when the recipient is with happy rather than sad facial expression. In contrast, when eye contact is absent, consumers are more willing to donate when the recipient is with sad rather than happy expression. In addition, emotional intensity shows significant mediated moderation effect.

### Theoretical Contributions

The findings from our study provide the following theoretical contributions. Firstly, given the ambivalent evidence on the relative effectiveness of happy and sad facial expressions, this study explains when happy (vs. sad) expression is more effective and when vice versa. Specifically, prior studies examining the effect of facial expressions (e.g., happy vs. sad) on charitable donation willingness provided conflicting results (Cao and Jia, 2017; Shepherd et al., 2018). In this study, we propose that donor-recipient eye contact is a potential moderator. Results from our study suggest that when eye contact is present, happy rather than sad facial expression of the recipient can result in higher donation intentions; however, in the absence of eye contact, sad facial expression is more effective than happy expression. Moreover, our study highlights the role of emotional intensity in the effectiveness of a charity advertisement. A majority of studies on donation focus on understanding donations of consumers from a cognitive perspective (Smith and McSweeney, 2007; Lin, 2021) and largely leave aside potential emotional responses of consumers. Although a few have examined donation behaviors from an emotional perspective (Yarkoni et al., 2015; Urbonavicius et al., 2019), these studies generally discuss the role of different types of emotions and provide few insights into the role of emotional intensity. We extend the existing research by examining the mediating role of emotional intensity. The results from our empirical study show that emotional intensity of consumers has significant mediation effects when eye contact is present and when it is absent. We contribute by offering more in-depth understandings of the mechanism underlying responses of consumers to a charity advertisement.

### Managerial Implications

This study can provide several managerial implications to help charity organizations improve the persuasiveness of a charity advertisement. Findings from our study can help managers of charity organizations to better design a charity advertisement and encourage consumers to make donations. In some circumstances, potential recipients or charity organizations may intentionally choose to cover the eyes of recipients in a charity advertisement for privacy concerns. The results from our study indicate that charity organizations can strategically choose the facial expression of recipients to enhance emotional intensity of donors, and, in turn, increase their donation intentions. In a charity advertisement, when the eye contact between donors and recipients is present, it may be more effective to choose to use happy facial expression in the advertisement. For instance, some organizations may intentionally cover the eyes of recipients to protect their privacy, and, in such a case, a charity advertisement may be more effective when managers choose to use sad rather than happy expressions. However, in conditions when eye contact between a donor and a recipient is present, consumers tend to have approach motivation and are more likely to be persuaded by happy rather than sad facial expression. In summary, the results of our study can help managers of charity organizations understand the psychological mechanism underlying donation behaviors of consumers and provide practical implications on charity advertisement design.

### Limitations and Future Research Directions

Admittedly, our study has a few limitations that need to be addressed for future research. First, photos of a child were used as stimuli in the two experiments of this study. Although a children-related charitable advertisement usually appeals an extensive social support (Polonsky and Grau, 2008; Bekkers and Wiepking, 2011), children may have significant difference compared with adults, such as face shape, which prior research showed may have significant influence on donation intention (Zebrowitz et al., 2007). Thus, researchers can consider examining the interaction effect of facial expression and eye contact, using photos of different adults or different children. Moreover, we focus on examining the interaction of facial expression and eye contact on donation intentions, and have not fully considered potential impacts of the demographic characteristics of recipients, such as gender (Fink et al., 1975), age (Paulson, 2014), face shape (Zheng et al., 2018). Considering that these characteristics might have influence on the effectiveness of a charity advertisement, we encourage future research may consider investigating the potential impacts of these factors.

## Data Availability Statement

The raw data supporting the conclusions of this article will be made available by the authors, without undue reservation.

## Ethics Statement

The studies involving human participants were reviewed and approved by MBA Education Centre of Hainan University. Written informed consent from the participants’ legal guardian/next of kin was not required to participate in this study in accordance with the national legislation and the institutional requirements.

## Author Contributions

MY and JZ collected the literature. ZT built the theoretical framework and proofread the manuscript. WF designed the experiments. YY collected the data. DL organized the manuscript. All authors contributed to the article and approved the submitted version.

## Conflict of Interest

The authors declare that the research was conducted in the absence of any commercial or financial relationships that could be construed as a potential conflict of interest.

## Publisher’s Note

All claims expressed in this article are solely those of the authors and do not necessarily represent those of their affiliated organizations, or those of the publisher, the editors and the reviewers. Any product that may be evaluated in this article, or claim that may be made by its manufacturer, is not guaranteed or endorsed by the publisher.
